# Cytoskeletal Rearrangements in Synovial Fibroblasts as a Novel Pathophysiological Determinant of Modeled Rheumatoid Arthritis

**DOI:** 10.1371/journal.pgen.0010048

**Published:** 2005-10-28

**Authors:** Vassilis Aidinis, Piero Carninci, Maria Armaka, Walter Witke, Vaggelis Harokopos, Norman Pavelka, Dirk Koczan, Christos Argyropoulos, Maung-Maung Thwin, Steffen Möller, Waki Kazunori, Ponnampalam Gopalakrishnakone, Paola Ricciardi-Castagnoli, Hans-Jürgen Thiesen, Yoshihide Hayashizaki, George Kollias

**Affiliations:** 1 Institute of Immunology, Alexander Fleming Biomedical Sciences Research Center, Athens, Greece; 2 RIKEN Genomic Sciences Center, Yokohama, Japan; 3 Mouse Biology Programme, EMBL, Monterotondo, Italy; 4 Department of Biotechnology and Bioscience, University of Milano-Bicocca, Milan, Italy; 5 Institute of Immunology, University of Rostock, Rostock, Germany; 6 Laboratory of Medical Physics, University of Patras, Patras, Greece; 7 Department of Anatomy, National University of Singapore, Singapore; The Jackson Laboratory, United States of America

## Abstract

Rheumatoid arthritis is a chronic inflammatory disease with a high prevalence and substantial socioeconomic burden. Despite intense research efforts, its aetiology and pathogenesis remain poorly understood. To identify novel genes and/or cellular pathways involved in the pathogenesis of the disease, we utilized a well-recognized tumour necrosis factor-driven animal model of this disease and performed high-throughput expression profiling with subtractive cDNA libraries and oligonucleotide microarray hybridizations, coupled with independent statistical analysis. This twin approach was validated by a number of different methods in other animal models of arthritis as well as in human patient samples, thus creating a unique list of disease modifiers of potential therapeutic value. Importantly, and through the integration of genetic linkage analysis and Gene Ontology–assisted functional discovery, we identified the gelsolin-driven synovial fibroblast cytoskeletal rearrangements as a novel pathophysiological determinant of the disease.

## Synopsis

Rheumatoid arthritis (RA) is a chronic destructive disease that affects 1–3% of the general population, exacting substantial personal, social, and economic costs. Current treatments alleviate the symptoms and offer immediate relief for many patients but do not cure the disease. While the cause of the disease remains poorly understood, the completion of the Human Genome Project and the emergence of functional genomics and high-throughput technologies offer intriguing new possibilities. For example, expression profiling creates a molecular fingerprint of the disease status by quantifying the expression levels of thousand of genes simultaneously. Similarly, reverse genetics (the genetic modification of a particular gene in search of its function) allow for the creation of animal models of disease. To discover novel genes and/or cellular pathways involved in the development of the disease, the authors used two methods in an animal model of RA for large-scale expression profiling. They identified a large number of genes and molecular processes that are deregulated in the disease. Using this information, the authors described pathophysiologic determinants of RA and created a unique list of disease modifiers of potential therapeutic value.

## Introduction

Rheumatoid arthritis (RA) is a chronic destructive arthropathy with a prevalence of 1–3% and substantial personal, social, and economic costs. It is characterized by prolonged inflammation of the joints, eventually leading to destruction of the cartilage and bone. Inflammation is initially localized in the synovial lining, a monolayer of synovial cells that lines diarthroidal joints. In RA, the synovial lining becomes markedly thickened due to synovial cell proliferation and infiltration by inflammatory cells. This proliferative mass, the pannus, invades and destroys articular cartilage and bone, leading to irreversible destruction of joint structure and function [1]. Current therapies of RA rely mainly on symptomatic treatment with nonsteroidal antiinflammatory drugs and/or with disease-modifying antirheumatic drugs. However, even the best available treatments (such as targeting tumour necrosis factor [TNF] and TNF signalling) do not cure the disease and do not even sufficiently retard progression in the majority of the patients, while they often exhibit adverse side effects [2].

Despite intense efforts, the aetiology and pathogenesis of RA remain poorly understood. Traditional research paradigms for RA have implicated a variety of mechanisms that contribute to the initiation and perpetuation of synovial inflammation, including autoantibodies and immune complexes, T cell-mediated antigen-specific responses, persistence of cytokine networks and other proinflammatory molecules, genetic bias and sex predisposition, and tumour-like behaviour of the arthritic synovium [3]. Animal models of RA share many clinical features with the human disease and hence constitute valuable tools in deciphering the pathogenic mechanisms that govern disease activation and perpetuation [4]. Among them, the TNF-transgenic (TNF-Tg) mouse [5] has been instrumental in demonstrating the role of TNF in the development of the disease and foreshadowed the introduction and success of anti-TNF therapies that transformed the effective management of the disease [6]. In this model, chronic overexpression of human TNF results in a chronic, erosive, symmetric polyarthritis, with 100% phenotypic penetrance, timed disease onset, and progressive histological symptoms that closely resemble human RA [5–7].

To gain further insights into the pathophysiology of the disease and to discover genes and/or pathways involved in its pathogenesis, we have utilised the TNF-Tg animal model of RA for large-scale expression profiling with both subtractive libraries and oligonucleotide microarray hybridizations. Differential expression was validated by a number of methods, in both mouse and human patient samples, thus creating a unique database of potential disease modifiers and therapeutic targets. Moreover, in an attempt to discover deregulated cellular functions based on functional annotations of deregulated genes, we identified the gelsolin-driven synovial fibroblast cytoskeleton rearrangement as a pathophysiological determinant of the disease.

## Results

To discover genes and cellular pathways that participate in the pathogenesis of RA on a large scale, we used a twin high-throughput approach, comprising two entirely different methodologies governed by different constraints and analyzed by different statistics. Total RNA samples were extracted from whole-joint (WJ) and synovial fibroblast (SF) ex vivo cultures isolated from 6-wk-old mice with RA (Tg197, *hTNF* ^+/−^; disease severity index 3; [5]) and their normal (wild-type [WT]) littermates. Each sample (out of four: RA SF, WT SF, RA WJ, and WT WJ) consisted of equimolar amounts of RNA pooled from four (two male and two female) mice (16 mice altogether), to some extent equalizing biological diversity. Biological replicates (different extractions from independent mice, employing the same extraction protocols and pooling strategy) were used for both the creation of subtractive cDNA libraries and the hybridization of oligonucleotide microarrays.

### Subtractive cDNA Libraries and Large-Scale Sequencing

Four different full-length cDNA libraries (RA SF, WT SF, RA WJ, and WT WJ) were normalized and subtracted to each other, as outlined in Figure S1A. Due to experimental design, the resulting subtracted libraries (L0, L1, L2, and L7) contained cDNAs from the tester cDNA library only and therefore constituted libraries of up-regulated genes in the corresponding tester library (or of down-regulated genes in the driver library). From the subtractive libraries, 27,511 cDNAs/clones were sequenced and clustered to 9,176 clusters/genes, as summarized in Figure S1B. Each gene was then annotated through BLAST homology searches at Unigene, FANTOM, and SWISSPROT databases.

In summary, among the 9,176 genes found, 7,977 corresponded to known genes, while 1,199 had sequences not reported previously. Each gene was represented by a different number of clones in almost all libraries, directly proportional to subtraction efficiency and transcript abundance. The relative distribution of each gene in each library is the true measure of differential expression, which can be obscured by sampling errors arising by chance when the clones are selected. Therefore, in order to identify the truly differentially expressed genes, a likelihood value *(R)* was assigned to each gene from pairwise comparisons of the relative libraries (SF/L0L1 and WJ/L2L7). The statistical significant thresholds were then calculated (Figure S2), and two significance levels were selected (summarized in Table 1): a very high one (99.99%; *p* ≤ 0.0001) to report the results independently, and a lower one (99%; *p* ≤ 0.01) for comparison with the corresponding results from the DNA microarray hybridizations. Known (by a Unigene cluster ID) differentially expressed genes at 99.99% significance level are presented in Table S1.

**Table 1:**
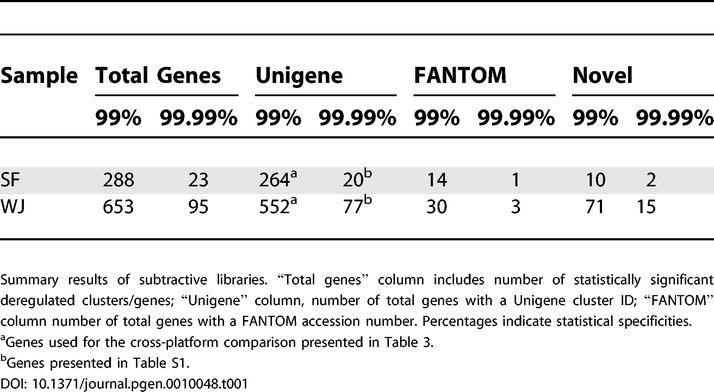
Summary Results of Subtractive Libraries and Microarray Hybridizations

**Table 2:**
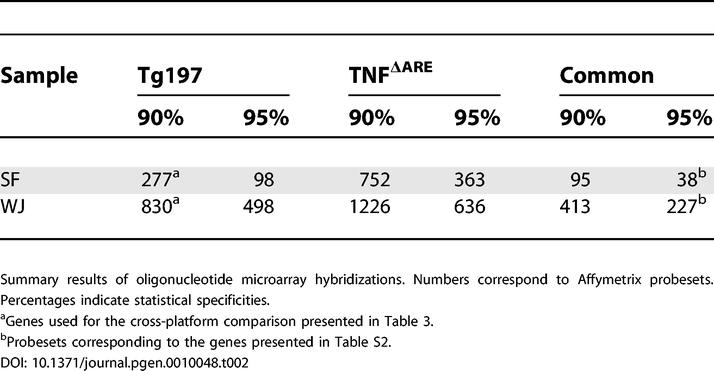
Summary Results of Oligonucleotide Microarray Hybridizations

**Table 3:**
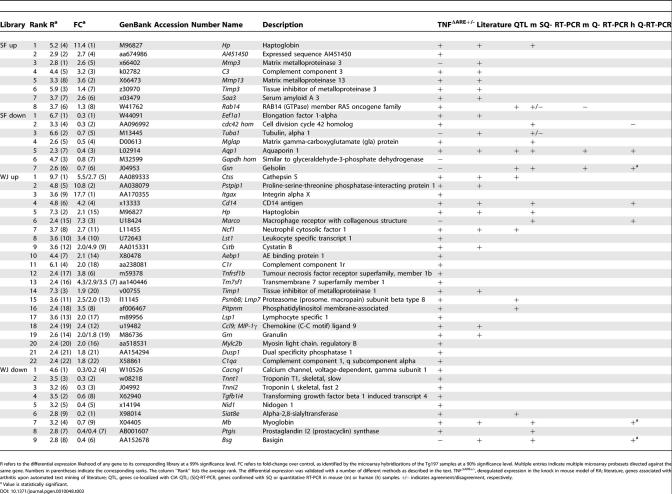
Differentially Expressed Genes in Arthritic WJs or SFs Commonly Identified by Subtractive Libraries and Oligonucleotide DNA Microarray Hybridizations

### Oligonucleotide Microarray Hybridizations

Fluorescently labeled cRNA probes made from similar (biological replicates, see above) samples of total RNA (RA SF, WT SF, RA WJ, and WT WJ) were utilized to hybridize in duplicate the Affymetrix Mu11K oligonucleotide DNA chipset (eight chipsets, 16 chips total). Furthermore, similar samples (equimolar amounts of RNA pooled from four—two male and two female—6-wk-old arthritic mice; severity index 3) of total RNA (from SF and WJ) from another animal model of arthritis (spontaneous, knock-in, *TNF* ^ΔARE+/-^) [8] were used for additional chip hybridizations (four chipsets, eight chips total). The MIAME-compliant [9] microarray data (see the ArrayExpress database [10], accession number E-MEXP-255) were normalized and analyzed as outlined in Figure S3 and described in detail in Materials and Methods and Figure S4. Differentially expressed genes (DEGs) were selected, utilizing a sample-specific fold-change model (FCM) [11] at different significance levels (90%, *p* ≤ 0.1; 95%, *p* ≤ 0.05), where selected DEGs have always an observed fold-change higher than the expected fold-change. The results for both animal models are summarized in Table 2. Sample-specific DEGs common to both animal models (significance: 95%, *p* ≤ 0.05) are presented in Table S2.

### Cross-Platform Comparison and Validation

Both differential expression analysis methods presented above produced lists of deregulated genes of high statistical significance. To validate the results independently and to avoid performing numerous RT-PCRs, differential expression results by both platforms of analysis were compared to each other for the same animal model (Tg197, *hTNF* ^+/-^), sample type (WJ, SF), and direction of deregulated expression (up or down). The comparison, at significance levels 99% for the libraries and 90% for the microarrays (selected based on similar output gene numbers), was performed through the NetAffx database (http://www.affymetrix.com). Although few examples of expression profiling cross-platform overlaps have been reported [12,13], in this study 46 genes (15 for SF, 31 for WJ) were commonly predicted as up- or down-regulated by both methods (combined *p*-value of 0.001) (Table 3).

To verify the validity of the twin high-throughput approach and to prove that the reported gene list is self-validated, a number of the predicted deregulated genes were confirmed with different methods. Expression profiling in the knock-in disease model (*TNF* ^ΔARE+/-^) confirmed 40 of the genes (Table 3). Automated literature text mining (Biolab Experiment Assistant, BIOVISTA, Greece) identified 20 of the genes previously associated with RA (Figure S5; summarized in Table 3). In addition, 11 representative genes (SF: *Gsn, Aqp1, mglap, cdc42 hom,* and *Hp;* WJ: *Marco, Hp, CD14, Mb, Bsg, Ptgis*) were further confirmed by semi-quantitative (SQ) RT-PCR (Figure S6A–S6C; summarized in Table 3). All SQ-RT-PCRs were performed in the linear range of the reaction (at three different concentrations normalized against housekeeping genes) in biological replicates (different extractions from independent mice, employing the same extraction protocols and pooling strategy) of the samples used for both the subtractive libraries and the microarray hybridizations. Representative genes were selected on the basis of (1) different sample source (six from WJs and five from SFs), (2) different direction and degree of deregulated expression (six up-regulated and five down-regulated), and (3) biological interest and potential follow-up. Moreover, two of them (SF: *Gsn, Aqp1*) were also confirmed by real-time RT-PCR (Figure S6D; summarized in Table 3).

**Figure 1:**
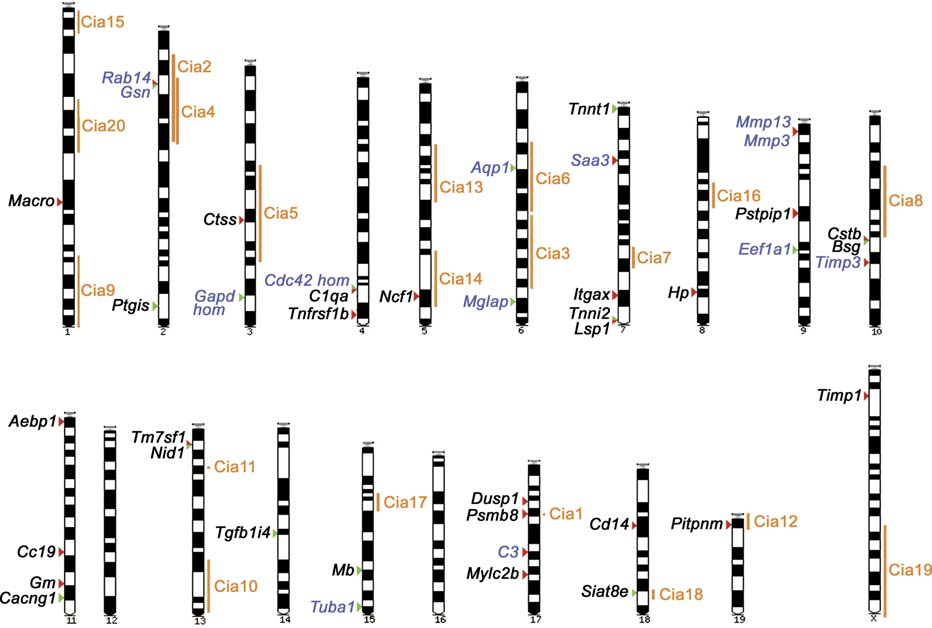
Chromosomal Localization of Identified Deregulated Genes in the RA Animal Model, Together with the QTL for CIA Red and green arrowheads indicate up- and down-regulated genes, respectively. Black and blue lettering refers to deregulated genes in WJs and SFs, respectively.

In an attempt to combine gene expression analysis with genetic linkage analysis, all differentially expressed genes were mapped to the chromosomes together with the known quantitative trait loci (QTL, chromosomal regions/genes segregating with a quantitative trait) for an induced animal model of arthritis, collagen-induced arthritis (CIA) [14]. As graphically represented in Figure 1 (and summarized in Table 3), eight genes mapped to CIA QTL (WJ: *Ctss, Pitpnm, Ncf1, Psmb8,* and *Siat8e,* SF: *Rab14, Aqp1,* and *Gsn*).

**Figure 2:**
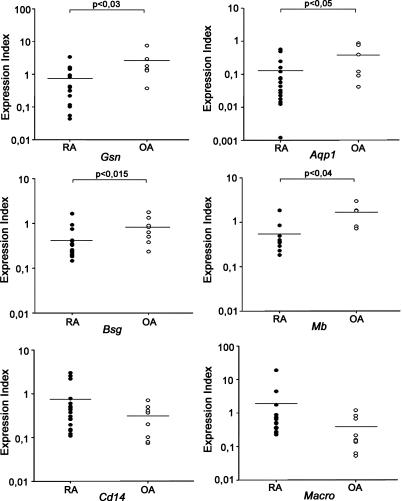
Confirmation of Deregulated Expression in Human Patient Samples Quantitative RT-PCR for the indicated genes for 19 RA and eight osteoarthritis (OA) samples. Values were normalized to the expression of *B2m* and were expressed as expression index. Similar results were obtained upon normalization to *L32*. Nonparametric Mann-Whitney statistical tests were used to derive *p*-values.

The expression level of seven of the genes found deregulated in the arthritic mice and confirmed by RT-PCR in mouse samples, was also examined in human patients' RNA samples with real-time RT-PCR analysis. Due to the lack of normal (WT) human synovium samples, we compared the expression of 19 RA samples with eight osteoarthritis samples as controls, a consensus strategy for differential expression analysis in arthritis [15,16]. As shown in Figure 2 and summarized in Table 3, the deregulated expression of six (out of seven assayed) of the genes was confirmed in humans as well, including four with high statistical significance *(Gsn, Aqp1, Bsg,* and *Mb),* thus extending the validity and utility of the mouse model-generated deregulated gene list to the human disease.

### Arthritic Synovial Fibroblasts Have a Rearranged Cytoskeleton

**Figure 3:**
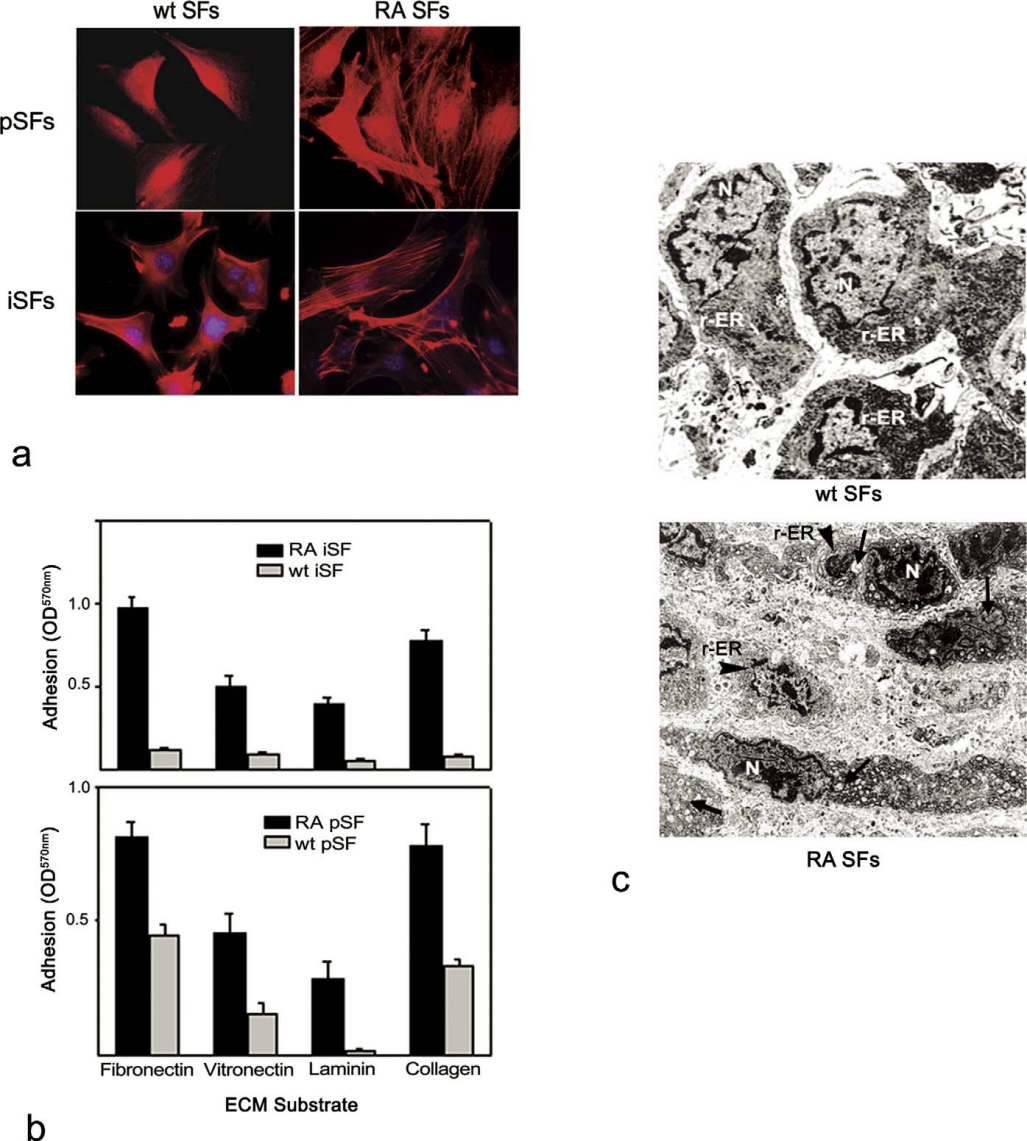
Mouse Arthritic SFs have a Rearranged Cytoskeleton and Increased ECM Adhesion (A) Immunofluoresence of arthritic (RA) or normal (WT) primary and immortalized synovial fibroblasts (pSFs and iSFs respectively) for F-actin. (B) Adhesion assays of arthritic (RA) or normal (WT) primary and immortalized SFs on purified ECM components as described in Materials and Methods. Error bars indicate standard deviation of triplicate samples from their mean value. (C) Transmission electron microscopy (TEM, magnification 5,000×) of SFs from ankle joints isolated from WT and arthritic mice. Arrowheads indicate dilation r-ER, while arrows point to swollen mitochondria with distorted cristae. N, nuclei.

The twin high-throughput expression profiling approach described above yielded a large number of disease-implicated, deregulated genes of high statistical significance. Furthermore, to (1) prove the validity and extend the utility of the expression data analysis even further, (2) infer deregulated biological functions from the gene expression data, and (3) define functional criteria for further gene selection, the selected genes were annotated in the form of the Gene Ontology (GO) [17] term “biological process.” GO term frequencies in the selected gene lists were then calculated, and their statistical significance was estimated. As shown in Table S3, predicted deregulated functions in SFs include, as expected, collagen catabolism, complement activation, and immune and stress responses. Interestingly, five out of 26 significantly (*p* < 0.01) deregulated GO functions concerned (directly or indirectly) the actin cytoskeleton, suggesting that arthritic SFs have a rearranged actin cytoskeleton. In order to confirm the prediction, F-actin was visualized in vitro on arthritic as well as WT SFs (both primary and immortalized) in mice. As is evident from Figure 3A, arthritic SFs exhibit pronounced stress fibers, thus validating the in silico, expression-based hypothesis.

Stress fibers within fibroblasts allow them to exert tension on the extracellular matrix (ECM) surrounding them—an essential process in wound healing. It is well understood that differences in the actin cytoskeleton reflect altered ECM attachment properties and/or vice versa. Indeed, arthritic SFs were shown to adhere to different proteins of the ECM (fibronectin, vitronectin, laminin, and collagen) with increased affinity in vitro (Figure 3B).

Attachment to the ECM (and associated cytoskeletal changes), mediated mainly through engagement and clustering of transmembrane integrin molecules, largely define cell shape and morphology, as well as their behavior and fate. Increased adhesion to the ECM is expected to lead to a more elongated shape. In order to confirm the increased adhesion of the arthritic fibroblasts in vivo, we examined ankle joints from arthritic or WT littermate mice on an ultrastructural level with transmission electron microscopy (Figure 3C). In WT mice, SFs contained prominent nuclei, abundant rough endoplasmic reticulum (r-ER), and mitochondria of different shapes and sizes. In contrast, remarkable modification of the SFs was noticed in the joints of the arthritic mice, where most randomly flattened cells had an elongated shape characterized by dilation of the r-ER and by swollen mitochondria with distorted cristae.

**Figure 4:**
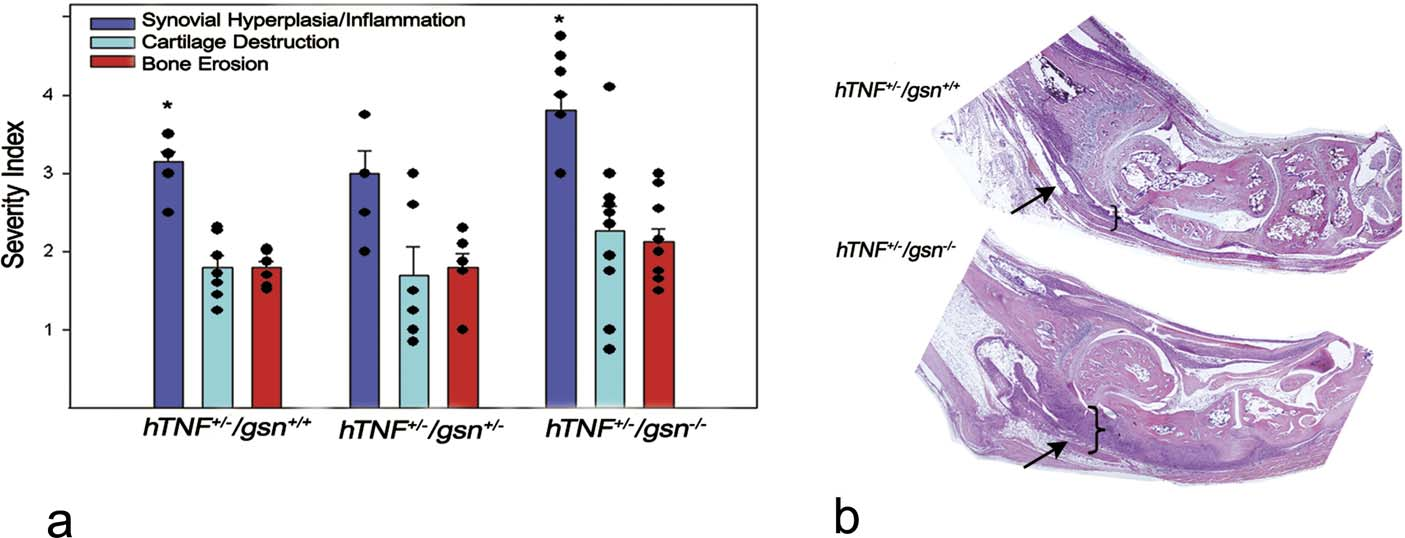
Knocking out Gelsolin Expression Results in Disease Exacerbation (A) Histopathological scores**.** * *p* = 0.025 *n* = 8–11. (B) Representative histopathological analysis (stained with haematoxylin-eosin) of arthritic joints. Shown images were assembled from multiple overlapping sections. Arrows indicate the synovial membrane.

Therefore, it seems that one of the pathogenic mechanisms in RA is the promotion of actin polymerization and rearrangement of the actin cytoskeleton. *Gelsolin (Gsn)* is a gene that maps in one of the CIA QTLs (see Figure 1), and its expression was found down-regulated in arthritic SFs by both subtractive libraries and microarray hybridizations (see Table 3). Its deregulated expression was confirmed by real-time RT-PCR in both mouse (see Figure S6D) and human samples (Figure 2), as well as by Western blot in immortalized SFs (see Figure S6E). The gene encodes an actin-binding protein with filament-severing properties [18], and *Gsn* ^−/−^ fibroblasts have excessive actin stress fibers [18], very similar to the ones observed in arthritic SFs (see Figure 3A). In order to prove the involvement of cytoskeletal organization in the pathogenesis of RA and to highlight the role of gelsolin in it, the arthritic mice (Tg197, *hTNF* ^+/−^) were mated with the *gsn* knockout mice (*gsn* ^−*/*−^) [18]. Knocking out *gsn* expression from SFs should promote RA pathogenesis by inhibiting the severing activity of gelsolin. Indeed, and as shown in Figure 4, abolishing *gsn* expression resulted in hyperplasia of the synovial membrane and exacerbation of the disease.

## Discussion

### Twin Expression Profiling, Statistical Analysis, and Validation

Expression profiling, the relative quantification of the expression levels of thousands of genes simultaneously, is one of the most promising approaches for understanding mechanisms of differentiation, development, and disease. However, the small number of samples usually employed substantially limits statistical analysis and precludes application of complex multivariate methods, which would be more appropriate for polygenic diseases such as RA. As a consequence, results should be confirmed by an independent biological method, which largely diminishes the high-throughput nature and discovery rate of any given approach.

To overcome these problems and increase the discovery rate, we used a twin high-throughput approach composed of subtractive cDNA libraries and oligonucleotide DNA microarray hybridizations. Both methodologies are governed by different constraints: random chance for the libraries' robotic clone selection and chip design for the microarrays. Consequently, both methodologies are completely uncorrelated towards the propagation of error. Therefore, the intersection of their statistically significant deregulated gene lists is expected to have a very low false discovery rate. To illustrate this and to exhibit the validity of the twin high-throughput approach, a number of representative genes from the commonly selected list was confirmed by a number of different methods, such as automated literature search and SQ and quantitative RT-PCR in both mouse and human samples. Therefore, we have shown that coupling two different high-throughput approaches largely decreases the need for independent confirmation and consequently increases the number of likely deregulated genes.

All differentially expressed genes were mapped to the chromosomes, together with the known QTL for an animal model of arthritis, CIA [14]. Eight deregulated genes mapped to CIA QTL (WJ: *Ctss, Pitpnm, Ncf1, Psmb8,* and *Siat8e;* SF: *Rab14, Aqp1,* and *Gsn;* see Figure 1), suggesting that these genes may have a dominant influence in arthritic processes, irrespective of the inciting stimulus (autoimmune or inflammatory). To that end, it was recently reported that a naturally occurring polymorphism of *Ncf1* (an NADPH oxidase subunit; QTL Cia 14) regulates arthritis severity [19] and is currently being commercially exploited in a drug discovery programme. Similarly, knocking out *Aqp1* (which encodes a water channel protein; QTL Cia 6) expression revealed its fundamental role in cell migration—central to wound healing and tumour spread [20]. Interestingly, several of the genes cluster together in adjacent regions of the chromosomes (4, 7, 10, 11 and 13). These loci could define new QTL or imply common regulatory control at the chromatin level.

### Actin Cytoskeleton and RA Pathogenesis

For polygenic diseases such as RA, knowledge about concerted gene functions or cellular processes might provide valuable clues and help to prioritize targets. In this context, we searched for deregulated processes rather than genes, based on functional annotations of deregulated genes, as these are formalized through the controlled vocabulary of the Gene Ontology Consortium [17]. GO term frequencies were calculated in the selected gene list and their statistical significance was estimated. The resulting list (Table S3) includes a number of expected functions that encompass accumulated knowledge about the pathogenesis of the disease, such as collagen catabolism, complement activation, and immune and stress responses. More importantly, five out of 26 predicted deregulated GO functions concerned (directly or indirectly) the actin cytoskeleton, thus forming a valid hypothesis to be explored. Accordingly, F-actin stress fibers were visualized in vitro, in arthritic and WT SFs, both primary and immortalized. As is evident in Figure 3A, there was a striking difference, with the arthritic SFs exhibiting pronounced stress fibers. Notably, stress fibers appear in differentiated fibroblasts called myofibroblasts, specialized contractile fibroblasts with an important role in establishing tension during wound healing and pathological contracture [21,22]. This is the first direct indication of the possible presence of myofibroblasts in the arthritic synovium, although it has been previously reported that transforming growth factor-b1 and interleukin-4 can induce a myofibroblastic phenotype to SFs in vitro [23]. Consistent with this notion, we observed that arthritic SFs have more pronounced focal adhesion kinase-positive islands (unpublished data), a prominent feature of myofibroblasts [22]. The actin cytoskeleton interacts bidirectionally with the ECM through receptors (mainly integrins) that possess extracellular binding sites for laminin, collagen, fibronectin, and other ECM components. The formation of intimate, extensive adhesive contacts between cells and ECM results from cooperation between adhesive systems and the actin cytoskeleton and the generation of force across regions of the cell [24]. In this context, myofibroblasts are thought to exert increased tension to the substratum through their increased adhesive capacity, which results in a more flattened, elongated cell shape [22]. Accordingly, we have observed that arthritic SFs adhere to various ECM components with increased affinity in vitro (see Figure 3B), resulting in a more elongated shape in vivo (see Figure 3C), further corroborating the possible existence of myofibroblasts in the arthritic synovium. In accord with this result, the possible presence of myofibroblasts in the human arthritic synovium was recently implied, by immunohistochemical analysis [25].

While the existence of myofibroblasts and their role in the pathogenesis of RA remain to be further explored, the fact remains that the reorganization of the actin cytoskeleton and the associated deregulation of ECM adhesion seem to be an intrinsic property of arthritic SFs. To this end, it was recently reported that the prevalence of specific autoantibodies against cytoskeletal antigens is elevated in patients with RA [26]. Autoantibodies serve as important serological markers in the diagnosis of various autoimmune and connective tissue diseases, including RA [27,28]. A large number of RA-specific autoantibodies of high diagnostic value are directed against components of the cytoskeleton: anti-vimentin, anti-keratin, and anti-filaggrin [28]. Filaggrin is a keratin cross-linker, an intermediate filament-aggregating protein, that can affect other cytoskeletal elements, including actin microfilaments, by a mechanism similar to actin filament severing by gelsolin [29]. Most of the above-mentioned autoantibodies recognize the citrullinated form of cytoskeletal proteins [30]. Since citrullination of proteins is not specific for RA, our results may provide the molecular basis, by a yet unknown mechanism, for the presence of anti-cytoskeleton antibodies in RA.

Recent experiments have shown that the cytoskeleton plays a critical role in the regulation of various cellular processes linked to cell transformation and tumorigenesis, such as contact inhibition and anchorage-independent cell growth [31]. Accumulated evidence suggesting that arthritic SFs also exhibit characteristic of transformed cells led to the working hypothesis that the arthritic synovium is a locally invasive tumour [32]. The rearranged cytoskeleton in arthritic SFs therefore reinforces the concept of a transformed-like character of the SF and opens up new directions in the pharmacological treatment of RA.

Gelsolin is an actin-binding protein [33] that has been implicated, among others, in the transduction of signals into dynamic rearrangements of the cytoskeletal architecture. In the presence of calcium, gelsolin severs preexisting actin filaments and caps them, thereby preventing monomer addition to their fast-growing ends. The barbed end cap is highly stable, even in the absence of calcium, unless displaced by interactions with regulatory phospholipids such as phosphatidylinositol-4,5-bisphosphate. In the presence of a large pool of profilin (another actin-binding protein), or under depolymerizing conditions, these gelsolin-capped ends allow the disassembly of populations of actin filaments by subunit loss from the pointed ends [34]. *Gsn* ^−*/*−^ fibroblasts were found to have excessive stress fibers in vitro [18], similar to the ones observed in arthritic SFs, where gelsolin was found to be down-regulated by a variety of methods. Knocking out *Gsn* expression from the arthritic mice resulted in exacerbation of the disease (Figure 4), therefore proving the participation of the actin cytoskeleton rearrangement in the pathophysiology of the disease. Extending the similarities of the arthritic synovium with tumours, gelsolin was found to be one of the most strikingly down-regulated markers upon malignant transformation of fibroblasts by Ras [35], while overexpression of a gelsolin mutant was shown to suppress Ras-induced transformation [36]. Its expression was undetectable or reduced in a majority of human gastric, bladder, lung, colon, and breast tumours [37–39].

### Conclusion

We have shown that the combination of sequencing of subtractive cDNA libraries and microarray expression analysis is highly reliable and yields self-validated targets and, when integrated with other functional genomics approaches such as genetic linkage and GO-assisted functional discovery, can provide novel insights into the pathophysiology of RA. As an example, we have investigated one of the predicted deregulated cellular processes, cytoskeletal organization, and one of the predicted deregulated genes involved in these processes, gelsolin, to show that gelsolin-driven actin cytoskeleton rearrangement is a novel pathophysiological determinant of RA.

## Materials and Methods

### Animals.

All mice were bred at the animal facilities of the Alexander Fleming Biomedical Sciences Research Center under specific pathogen-free conditions, in compliance with the Declaration of Helsinki principles. Mice were housed at 20–22 °C, 55 ± 5% humidity, and a 12-h light-dark cycle; water was given ad libitum. “Arthritic” transgenic mice (Tg197, *hTNF ^+/−^* maintained on a mixed CBA × C57BL/6 genetic background for over 20 generations), carried the human TNF gene where the 3′ UTR was replaced by the corresponding one from b-globin [5]. “Arthritic” knockin mice (*TNF ^ΔARE/+^* maintained on a 129/C57BL6 background for over 20 generations) expressed the endogenous *mTNF* gene, where 69 bp encompassing the *TNF* ARE (AU-rich elements) at the 3′ UTR have been deleted, resulting in increased message stability and translational efficiency [8].

### Cell isolation and culture.

SFs (VCAM^+^) were isolated from 6- to 8-wk-old mice essentially as previously described [7]. Fibroblasts were selected by continuous culturing for at least 21 d and a minimum of four passages. No macrophage markers could be detected by FACS analysis. Cells were grown at 37 °C, 5% CO_2_ in complete DMEM medium (Gibco-BRL, San Diego, California, United States) supplemented with 10% FBS and 100 U/ml of penicillin-streptomycin. The creation and culture conditions (33 °C, 10 U/ml of murine rIFN-γ) of conditionally immortalized SF cell lines has been described previously [7].

### RNA extraction.

Total RNA was extracted from subconfluent (70–80%) cultured SFs (primary or conditionally immortalized) with the RNAwiz reagent (Ambion, Austin, Texas, United States), followed by single passage through an RNeasy column (QIAGEN, Hilden, Germany) according to manufacturers' instructions. Total RNA was extracted from WJs using the guanidinium isothiocynate-acid phenol protocol [40], followed by a single passage through an RNeasy column. RNA integrity was assessed by electrophoresis on denaturing 1.2% agarose-formaldehyde gels. RNA quantity and quality were calculated based on OD readings at 260/280 nm.

### Generation of full-length cDNA libraries.

First-strand cDNA synthesis was performed using SUPERSCRIPT II (Life technologies/Invitrogen) at 56 °C in the presence of trehalose and sorbitol. The cap structure at the 5′ end of mRNAs was biotinylated, and full-length cDNAs were selected, after RNAse I treatment, using streptavidin-coated beads [41]. Second-strand synthesis was primed by the single-strand linker ligation method (SSLLM), where a double-stranded DNA linker (with random 6-bp, dN6 or dGN5, 3′ overhangs) is ligated to the single-stranded cDNA [42]. The second strand is made by primer extension using mixtures of long-range thermostable polymerases, followed by restriction digestion (BamHI and XhoI) and ligation to the λ-FLCI phagemid vector [42,43]. After packaging, cDNA libraries were amplified on solid phase, as previously described [44].

### cDNA library normalization and subtraction.

The procedure is outlined in Figure S1A. Amplified phagemid λ-FLCI cDNA libraries were used to infect the Cre-expressing bacterial cell line BNN-132 and the excised plasmids were isolated [43]. Double-stranded plasmid DNA was nicked by the site-specific (f1 origin) endonuclease GeneII and converted to circular single-stranded form by digestion with exonuclease III [44]. Circular, single-stranded plasmid cDNA libraries (tester libraries) were then subjected to a single normalization-subtraction step, with PCR-derived single strand antisense DNA drivers produced from these libraries [44]. Normalization refers to low CoT (reassociation rate) hybridization (CoT = 2) with drivers produced from the “self” library, and it aims to decrease the representation of highly expressed mRNAs. Subtraction refers to high CoT hybridization (CoT = 100~200) with drivers produced from different libraries, and it aims to remove mRNAs common in both populations. Hybridized double-stranded cDNAs were removed with two passes through a hydroxyapatite column. Nonhybridized, single stranded cDNAs were converted to double stranded and were subsequently electroporated into DH10b bacterial cells, where only tester cDNAs are able to propagate (due to the presence of a replication origin and antibiotic resistance) [44].

### High-throughput sequencing and sequence analysis.

Colony picking, cDNA sequencing and sequence analysis were performed essentially as previously described [45–47]. Sequences were filtered for primer and vector sequences [47] and masked for rodent-specific and mammalian-wide repeats with RepeatMasker (http://www.phrap.org). EST clustering was performed with stackPACK and d2_cluster (word size 6, similarity cutoff 0.98, minimum sequence size 50, window size 200) [48,49]. Homology searches with known genes were performed with BLAST [50,51] in the Unigene (http://www.ncbi.nlm.nih.gov/UniGene), FANTOM2 (http://fantom2.gsc.riken.go.jp) [52], and SWISSPROT (http://www.ebi.ac.uk/swissprot/ ) databases. BLAST results were associated with GO terms (http://www.geneontology.org) at http://source.stanford.edu/cgi-bin/sourceSearch (for Unigene), ftp://ftp.ebi.ac.uk/pub/databases/GO/goa/ SPTR/gene_association.goa_sptr.gz (for SWISSPROT), and http://fantom2.gsc.riken.go.jp (for FANTOM2). The detailed results (including clone numbers, clusters/genes, BLAST results and E values, accession numbers, and GO assignments) can be found in the corresponding author's Web site, at http://www.fleming.gr/en/investigators/Aidinis/data.html. Most of the sequencing data, have been already submitted to public databases [46], in the context of the ongoing FANTOM (Functional annotation of the mouse) project [52,53].

### Differential gene expression statistical analysis of subtractive libraries.

The differential gene expression/abundance among the different subtracted cDNA libraries was calculated with a single statistical test designed especially for this purpose [54]. Essentially, the formula is the entropy of a partitioning of genes among cDNA libraries and is described by an *R*-value, which is the log likelihood ratio statistic, that follows (2*R_j_*) an asymptotic χ^2^ distribution [54]. The formula for the statistic *R_j_* for the j^th^ gene is given by the expression:


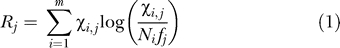


where


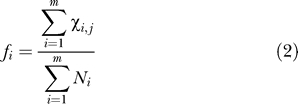


where *m* is the number of cDNA libraries, *N*_i_ is the total number of clones sequenced in the i^th^ library, *χ_i,j_* the counts (transcript copies) of the j^th^ gene in the i^th^ library, and *f_i_* is the frequency of gene transcripts copies of the j^th^ gene in all the libraries.

The significance of the *R*-statistic was established by utilizing a resampling method that tries to establish an “optimal” cutoff value using simulated library datasets based on the observed counts. The method consists of the following steps. (1) For every gene, the common gene transcript frequency *f_j_* is calculated with the help of Equation 2. This number corresponds to the expected frequency of gene transcripts under the null hypothesis of no difference in the abundance of gene j across all libraries. (2) The parameters of the Poisson distributions giving the sampling distribution of clone abundance for each gene and library are calculated under the null hypothesis. This parameter is equal to *N_i_* × *f_j_* for gene j in library i, where *N_i_* is the total number of clones (taking into account all genes) sequenced in library i and *f_j_* was calculated in step 1. This value is equal to the expected absolute number of clones of the j^th^ gene in the i^th^ library under the null hypothesis. (3) Then, for each library compared, and each gene, a random number is generated from the Poisson distribution of step 2. This number is a simulated count compatible with the null hypothesis, i.e., that the gene frequency for a particular gene is the same across all libraries. This step uses the random number-generating function of the computer to create an artificial dataset corresponding to the actual experiment of library creation and analysis for each of the libraries compared under the null hypothesis. (4) For gene j in the artificial dataset of step 3, the test statistic R is calculated by substituting for *χ_i,j_* the random number generated in step 3, for *f_j_* the frequency calculated in step 1, and for *N_i_* the total number of clones sequenced. (5) The *R*-values calculated in step 4 are sorted in descending order, and for a range of values for specificity (i.e., the true negative rate), the corresponding *R*-value is found. These are by definition true negatives, since they were obtained from libraries under the null hypothesis. (6) Steps 3–5 are repeated 1,000 times. The resulting data allowed us to construct the histogram shown in Figure S2A. These histograms depict the distribution of the *R*-value as a function of the required true negative rate cutoff. The mean of this (empirical) distribution is used as the *R*-value cutoff for the analysis of the experimental dataset. (7) The experimental dataset *R*-values are computed for all the comparative libraries using the observed clone counts. Genes with *R*-values greater than the previously established cutoff are considered to be differentially regulated between the libraries. Calculations were performed in the Computer Algebra System Mathematica 4.2 (http://www.wri.com) with the MathStatica add-on [55].

### High density oligonucleotide array hybridization.

cRNA probes were generated from 5 μg of total RNA and hybridized to the Mu11K (subunits A and B) chip set according to the manufacturer's instructions (Affymetrix, Santa Clara, California, United States) and as previously described [7]. The chip set is designed to collectively recognize 13,179 distinct murine transcripts, where the expression level of any given gene is interrogated by 40 oligonucleotides, 20 with a perfect match sequence and 20 carrying a single mismatch. Hybridized chips were washed and stained in a Fluidics Station (Affymetrix) following standard protocols; subsequently, fluorescence intensities were read by an Affymetrix scanner. All MIAME-compliant microarray data can be downloaded from the ArrayExpress database (accession number E-MEXP-255), as well as from the corresponding author's web site (http://www.fleming.gr/en/investigators/Aidinis/data.html).

### Microarray data preprocessing and normalization.

The procedure is outlined in Figure S3. Low-level analysis of the resulting scanned image (as background subtraction and computation of individual probe cell intensities) was automatically performed by MicroArray Suite 5.0 (MAS5) software (Affymetrix). Briefly, the quantitative level of expression (signal value), as well a qualitative measure of expression (detection call), of any given gene is calculated by proprietary Affymetrix algorithms from the combined, background-adjusted hybridization intensities of 20 perfect match and 20 single mismatch oligonucleotides (probe set). All signal values from all chips in the experiment were scaled to reach target intensity (TGT) of 500 or 2,500, following Affymetrix recommendations for the individual chip sets used. Scaled values were then submitted to a normalization step that is intended to force each chip subunit's signal distribution to have an identical overall shape across chips of the same subunit. Gene expression values were reorganized into two distinct data matrices, one for each chip set subunit (A,B), where rows and columns represented genes and chips, respectively. The columns of the data matrix were normalized to have the same quantiles using BioConductor [56]. Each quantile of each column was set to the mean of that quantile across arrays (see Figure S4A). The rows of the two data matrices were then merged, and the columns of the resulting data matrix were split according to the corresponding animal model of RA (Tg197, TNF^ΔARE^) and to the type of sample (SF or WJ), thus obtaining a total of four distinct data matrices. Finally, genes that were not called “present” (detection call) or that had a normalized signal lower than one-fifth of the TGT in at least one sample were not further considered. Subsequent analysis was therefore performed on the remaining 8,021 probe sets.

### Microarray data analysis.

Comparisons of Affymetrix chips hybridizations and real-time PCR have indicated that chip analyses are accurate and reliable, and that they underestimate differences in gene expression [57]. Nevertheless, in order to assess the system's performance, the reproducibility of the (technical) duplicate samples was examined, in terms of Pearson correlation coefficient of normalized signals, as well as detection concordance (expressed as the percentage of probe sets that were consistently detected with a “present” or an “absent” call in both chips). As can be seen in Figure S4B, Affymetrix data were highly reproducible and reliable.

As previously reported [58,59], the significance of fold-changes, which are commonly used as a measure of differential expression, is highly dependent on the expression level of the corresponding genes. Therefore, in order to identify genes that are significantly differentially expressed, we decided not to use a fixed fold-change threshold, but instead to model intensity-dependent fold-changes between replicated chips using a variant of a previously described approach [60]. Briefly, we first calculated fold-changes in pairwise comparisons of replicated chips representing the transcriptome of the same sample type (SF or WJ) measured (under the same experimental condition using the following definition:


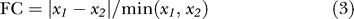


where *x*_i_ was the normalized expression level of a given gene in the i^th^ chip. Genes were then ranked according to the minor of their two expression values—i.e., min*(x_1_, x_2_)*—and the overall expression range was partitioned into ten intervals, each containing an equal number of probe sets. In each partition the median of all min*(x_1_, x_2_)* values as well as the 90^th^ and the 95^th^ percentile fold-change were determined, thus obtaining ten distinct modeling points for each sample type (SF or WJ) and for each of the two significance levels (90% or 95%). Based on the observation that measurement variability of high-density oligonucleotide microarrays depends on signal intensity following a power law [11], a continuous FCM was derived from the ten modeling points using a least squares linear fit in log-log plots. The following modeling parameters have been obtained for the two FCMs (see Figure S4C): a slope of −0.327 and an intercept of 2.356 for the SF FCM, and a slope of −0.346 and an intercept of 2.678 for the WJ FCM. This means that WJs are, as expected, intrinsically more variable than SFs. Using slopes (a90% or a95%) and intercepts (b90% or b95%) of the resulting regression curves, we could obtain for each given sample type (SF or WJ) and for any given minimum expression level both a 90% and a 95% significance threshold:


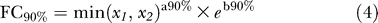



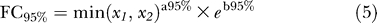


For each of the four data matrices (i.e., Tg197/SF, TNF^ΔARE^/SF, Tg197/WJ, and TNF^ΔARE^/WJ), an observed average fold-change between experimental conditions (diseased or normal) was calculated for each gene in the following way:


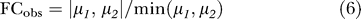


where μ*_i_* is the average expression level of a given gene in the i^th^ experimental condition. If no replicates were available, the single expression value of the given gene was used instead of the average. For each gene, this observed fold-change was then compared to the fold-change that was expected at a 90% (or 95%) significance level using the corresponding “sample type”-specific FCM, given the observed values of *μ_i_* of that gene:


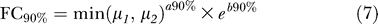



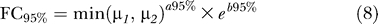


Finally, genes with FC_obs_ > FC_90%_ (or > FC_95%_) were selected as differentially expressed at a significance level of 90% (or 95%, respectively).

### Visualization of QTL and gene-expression data.

This was performed with Expression view [61] at http://ensembl.pzr.uni-rostock.de/Mus_musculus/expressionview. QTL data were derived from Serrano-Fernandez et al. [62].

### Functional clustering and determination of statistical significance.

Biological annotation, in the form of GO “Biological process” term [17], for each of the genes (probe sets) in Table 3, was obtained from the NetAffx portal (http://www.affymetrix.com). We then calculated the observed GO term frequencies, as means to discover deregulated functions (Table S3). The statistical significance of GO term frequencies was determined essentially as has been previously described [63]. Briefly, the hypergeometric distribution was used to obtain the chance probability of observing a given number of genes annotated in NetAffx with a particular GO term and then calculating appropriate *p*-values. More specifically, the probability of observing at least *k* probe sets annotated with a particular GO term within a list of selected probe sets of size *n* was calculated [63] as:


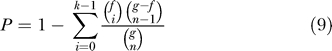


where *f* was the total number of genes within a functional category, and *g* was the total number of probe sets on the chip set (13,179).

### Adhesion assays.

These assays were performed on Cytometrix adhesion strips (Chemicon, Temecula, California, United States) coated with human fibronectin, vitronectin, laminin, and collagen I, according to the manufacturer's instructions. Briefly, cells (in triplicates) were allowed to adhere to the above-mentioned substrates, and unbound cells were removed with sequential washes with PBS containing Ca^++^ and Mg^++^. Adhered cells were then stained with crystal violet, solubilized, and their absorbance determined at 570 nm.

### Immunofluorescence.

Cells were fixed using 4% paraformaldehyde in PBS and stained by standard methods. For visualizing F-actin cells were stained with Alexa_594_-phalloidin (Molecular Probes, Eugene, Oregon, United States). P-tyrosine was detected using the 4G10 antibody (Upstate Biotechnology, Waltham, Massachusetts, United States), focal adhesion kinase was detected using a monoclonal antibody (clone 77, BD Transduction Laboratories, Lexington, Kentucky, United States). Antibodies against gelsolin were raised by immunizing rabbits with recombinant mouse gelsolin; immune serum was used at 1:500 dilution. For Western blot analysis, 10 μg of total cell protein was separated by SDS-PAGE, transferred to Immobilon-P membrane, probed for gelsolin, and re-probed for actin as an internal loading control.

### RT-PCR.

Total RNA was extracted from SF and WJ tissue using TRIzol reagent (Life Technologies, Rockville, Maryland, United States) in accordance with the manufacturer's instructions. RNA yield and purity were determined spectrophotometrically at 260/280 nm. First-strand cDNA synthesis was performed using the MMLV reverse transcriptase and oligo-dT_15_ (Promega, Madison, Wisconsin, United States).

SQ PCR was performed by 20–25 cycles of denaturation at 95 °C for 30 s, annealing at 57–62 °C (depending on the T_m_ of each individual set of primers) for 30 s, and extension at 72 °C for 1 min, in a final volume of 20 μl. The products were separated by electrophoresis on 1.5% agarose gel and stained with ethidium bromide. Product intensity was quantified with GelWorks 1D Advanced (v. 4.01) and normalized to the intensity of B2m and/or L32. The primers were selected to span two exons, while the two control primers were chosen from the Primer Bank database (http://pga.mgh.harvard.edu/primerbank/). Primer sequences (listed in the 5′ to 3′ direction, and designated as s, sense, and as, antisense) and product sizes (in bp) were as follows: *Aqp1* (s, TCACCCGCAACTTCTCAAAC; as, AGCTCTGAGACCAGGAAACA, 400), *Bsg* (s, ATGAGAAGAGGCGGAAGCCA; as, CCACTCCACAGGGCTGTAGT, 426), *Cd14* (s, CAATCCTGAATTGGGCGAGA; as, CGAGTGGGATTCAGAGTCCA, 400), *Cdc42* (s, AAGTGGCCCAGATCCTGGAA; as, AGCACTGCACTTTTGGGGTT, 380), *Gsn* (s, TGCAGGAAGACCTGGCTACT; as, ATGGCTTGGTCCTTACTCAG, 300), *Hp* (s, GAAGCAATGGGTGAACACAG; as, GGGGTGGAGAACGACCTTCT, 331), *Marco* (s, CACAGGAATTCAAGGACAGA; as, ATTGTCCAGCCAGATGTTCC, 397), *Mglap* (s, CAGTCCCTTCATCAACAGGA; as, CTGCAGGAGATATAAAACGA, 274), *Mb* (s, TCACACGCCACCAAGCACAA; as, TGGGCTCCAGGGTAACACTG, 354), *Ptgis* (s, TCACAGATGACCACACTCCC; as, GCAGTAGGACGACAAATTGT, 403), *B2M* (s, TTCTGGTGCTTGTCTCACTGA; as, CAGTATGTTCGGCTTCCCATTC, 104), and *L32* (s, TTAAGCGAAACTGGCGGAAAC; as, TTGTTGCTCCCATAACCGATG, 100).

Quantitative real-time PCR was performed using the iCycler iQ Real-Time detection system and the IQ SYBR Green Supermix (Bio-Rad Laboratories, Hercules, California, United States), according to the manufacturer's instructions, for one cycle of 94 °C for 4 min, and 40 cycles of denaturation at 95 °C for 50 s and annealing at 57–62 °C for 50 s. Primers were chosen from exons separated by large introns, and the PCR quality and specificity was verified by melting curve analysis and gel electrophoresis. Values were normalized to B2m and/or L32 (using the same primers as for the SQ PCR). Mouse (m) and human (h) primer sequences and expected lengths were as follows (listed in the 5′ to 3′ direction, and designated as s, sense, and as, antisense): *mAqp 1* (s, TCACCCGCAACTTCTCAAAC; as, TCATGCGGTCTGTGAAGTCG, 123), *mGsn* (s, TGCAGGAAGACCTGGCTACT; as, TCGATGTACCGCTTAGCAGA, 130), *hAqp 1* (s, CTCCCTGACTGGGAACTCG; as, GGGCTACAGAGAGGCCGAT, 182), *hBsg* (s, TTCCTGGGCATCGTGGCTGA; as, GCGGACGTTCTTGCCTTTGT, 159), *hCd14* (s, CGGCGGTCTCAACCTAGAG; as, GCCTACCAGTAGCTGAGCAG, 142), *hCdc42* (s, AATTGATCTCAGAGATGACC; as, TTTAGGCCTTTCTGTGTAAG, 150), *hGsn* (s, GGTGTGGCATCAGGATTCAAG; as, TTTCATACCGATTGCTGTTGGA,199), *hMarco* (s, TGGGACGAGATGGAGCAAC; as, CCCTTAGTTCCAGTTTCCCCTT, 193), *hMb* (s, TTGGTGCTGAACGTCTGGG; as, CTGTGCCAGGGGCTTAATCTC, 249), *hB2M* (s, CTGAAAAAGATGAGTATGCC; as, ACCCTACATTTTGTGCATAA, 202) and *hL32* (s, TTAAGCGTAACTGGCGGAAAC; as, GAGCGATCTCGGCACAGTAA, 210).

Cycle threshold (Ct; the first cycle in which amplification can be detected) values were obtained from the iCycler iQ software for each gene of interest (GOI) and control housekeeping genes (HKG; L32 and/or b2m), together with amplification efficiencies (η; 80–120%). For the mouse samples, we calculated the relative expression of the samples to WT controls as reference samples using the gene expression-relative quantification Microsoft Excel add-on macro (Bio-Rad) that utilizes the following formulas: relative expression = 2^−(S ΔCt-R ΔCt)^, where ΔCt = GOI Ct − HKG Ct. For the human samples, Ct values were converted to concentration values (ng/ml) by utilizing the standard curve made by serial dilutions (in duplicates) of a reference sample. Values were normalized to the corresponding values of the reference (housekeeping) gene(s) and presented (in logarithmic scale for visualization purposes) as expression index.

### Arthritic score and histopathology.

Paraffin-embedded joint tissue samples were sectioned and stained with haematoxylin and eosin. Arthritic histopathology was assessed (in a blinded fashion) separately for synovial hyperplasia, existence of inflammatory sites, cartilage destruction, and bone erosion using a semiquantitative (0–5) scoring as described previously [64].

### Transmission electron microscopy.

Ankle joints (dissected from the right hind leg of each mouse—three Tg197 and three WT) were split open longitudinally through the midline between the tibia and the talus, and were pre-fixed with 2.5% glutaraldehyde in PBS (pH 7.4) overnight. After post-fixing with 1% osmium tetroxide in PBS for 2 h, the samples were dehydrated in a graded series of ethanol and processed into Araldite. Semithin sections (1.0 μ) were cut and stained with methylene blue to observe the orientation under the light microscope. Ultrathin sections (80–90 nm) were then cut with an ultramicrotome (Riechert-Jung Ultracut E, Leica, Wetzlar, Germany), mounted on copper grids, counterstained with uranyl acetate and lead citrate, and evaluated by electron microscope (CM120 Biotwin, FEI Company, Hillsoro, Oregon, United States).

## Supporting Information

Figure S1 Subtractive cDNA Libraries and Large-Scale Sequencing (A) Outline of experimental strategy for the preparation of subtracted cDNA libraries and analysis of differential expression.
(B) Summary of normalized and subtracted cDNA libraries and sequencing results.
(1.2 MB PDF)

Figure S2 Calculation of the Statistically Significant Thresholds of the *R*-Statistic at Different Specificities (A) Distribution of the *R*-statistic for the indicated specificities and pairwise library comparisons.
(B) Tabulated *R*-value cutoffs for the indicated specificities and pairwise library comparisons.
(1.4 MB PDF)

Figure S3 Microarray Data Normalization and Analysis Outline (775 KB PDF)

Figure S4 Microarray Data Normalization, Evaluation, and Statistical Selection (A) Quantile normalization on separated oligonucleotide chip subunits.
(B) Reproducibility of technical duplicate samples in Affymetrix hybridizations.
(C) “Sample type”-specific FCMs derived from replicated chips.
(2.2 MB PDF)

Figure S5 Deregulated Genes Previously Reported to Be Associated with RA The associations were identified through the Biolab Experiment Assistant text-mining software.
(A) PubMed identification numbers of corresponding publications.
(B) Schematic representation of text mining results. Red and green indicate up-regulated and down-regulated genes, respectively; black and blue indicate WJ and SF, respectively; numerical values indicate number of PubMed references.
(1.5 MB PDF)

Figure S6 Validation of Expression Profiling Results (A–C) SQ RT-PCR of the indicated genes from different cDNA amounts of arthritic or WT WJs, primary SFs (pSF) and immortalized SFs (iSF). Ethidium bromide-stained PCR products were quantified with GelWorks 1D Advanced (v. 4.01) software and were normalized against the expression of B2M.
(D) Real-time RT-PCR of the indicated genes from the indicated samples.
(E) Western blot of whole cell extracts (two independent preparations) from arthritic and WT immortalized SFs probed with antibodies to gelsolin and actin. Gelsolin immunostaining was quantified with GelWorks 1D Advanced (v. 4.01) software and normalized against the corresponding actin intensities.
(2.0 MB PDF)

Table S1 Differential Expression Results from Subtractive Libraries at 99.99% Specificity (32 KB XLS)

Table S2 Differentially Expressed Genes Common in Two Animal Models of RA from Oligonucleotide DNA Microarray Hybridizations at 95% Specificity (59 KB XLS)

Table S3 GO “Biological Process” Term Frequencies for the Differentially Expressed Genes in Arthritic SFs and WJs, Selected from Both Subtractive Libraries and Microarrays (24 KB XLS)

## Abbreviations

CIA: collagen-induced arthritis
DEG: differentially expressed gene
ECM: extracellular matrix
FCM: fold-change model
GO: gene ontology
RA: rheumatoid arthritis
r-ER: rough endoplasmic reticulum
SF: synovial fibroblasts
SQ: semiquantitative
Tg: transgenic
TNF: tumour necrosis factor
Q: quantitative
QTL: quantitative trait loci
WJ: whole joint
WT: wild-type
