# Correction: Cytoskeletal Rearrangements in Synovial Fibroblasts as a Novel Pathophysiological Determinant of Modeled Rheumatoid Arthritis

**DOI:** 10.1371/journal.pgen.0010073

**Published:** 2005-11-25

**Authors:** Vassilis Aidinis, Piero Carninci, Maria Armaka, Walter Witke, Vaggelis Harokopos, Norman Pavelka, Dirk Koczan, Christos Argyropoulos, Maung-Maung Thwin, Steffen Möller, Kazunori Waki, Ponnampalam Gopalakrishnakone, Paola Ricciardi-Castagnoli, Hans-Jürgen Thiesen, Yoshihide Hayashizaki, George Kollias

In *PLoS Genetics,* volume 1, issue 4, DOI: 10.1371/journal.pgen.0010048

The eleventh author's name was incorrectly given as Waki Kazunori; it should be Kazunori Waki.

