# Supplementary material for: Cytoskeletal Rearrangements in Synovial Fibroblasts as a Novel Pathophysiological Determinant of Modeled Rheumatoid Arthritis
Source: PLoS Genet. 2005 Oct 28;1(4):e48. doi: 10.1371/journal.pgen.0010048 (PMC1270006; doi:10.1371/journal.pgen.0010048)
Supplement: Figure S1 — (A) Outline of experimental strategy for the preparation of subtracted cDNA libraries and analysis of differential expression. (B) Summary of normalized and subtracted cDNA libraries and sequencing results. (1.2 MB PDF) [file pgen.0010048.sg001.pdf]

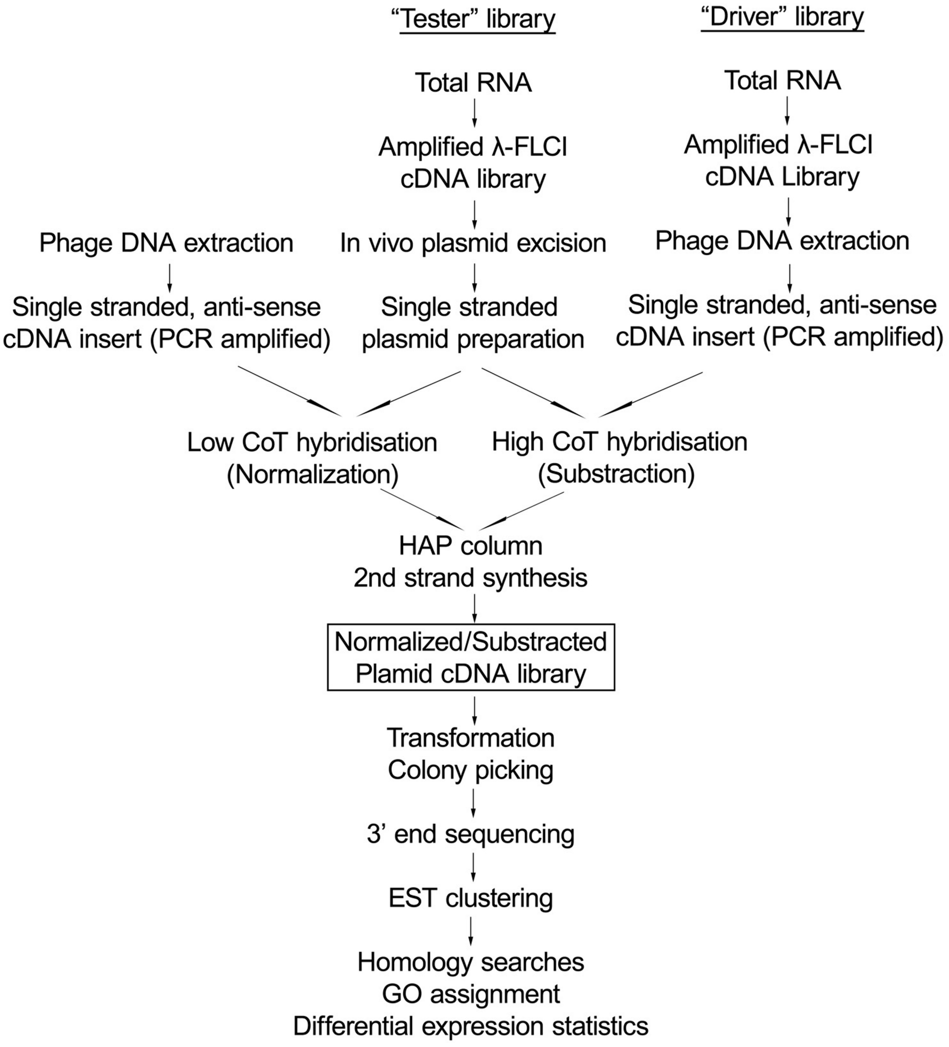

a

Subtracted library	Tester library	Driver library	Sequencing reads	Clusters
L0	RA SF	wt SF	7869	4097
L1	wt SF	RA SF	8602	4186
L2	wt WJ	RA WJ	7753	3331
L7	RA WJ	wt WJ	3287	1740
L0,L1,L2,L7			27511	9176*

b

Figure S1.

Subtractive cDNA Libraries and Large-Scale Sequencing

(A) Outline of experimental strategy for the preparation of subtracted cDNA libraries and analysis of differential expression.

(B) Summary of normalized and subtracted cDNA libraries and sequencing results.
