# Supplementary material for: Cytoskeletal Rearrangements in Synovial Fibroblasts as a Novel Pathophysiological Determinant of Modeled Rheumatoid Arthritis
Source: PLoS Genet. 2005 Oct 28;1(4):e48. doi: 10.1371/journal.pgen.0010048 (PMC1270006; doi:10.1371/journal.pgen.0010048)
Supplement: Figure S2 — (A) Distribution of the R-statistic for the indicated specificities and pairwise library comparisons. (B) Tabulated R-value cutoffs for the indicated specificities and pairwise library comparisons. (1.4 MB PDF) [file pgen.0010048.sg002.pdf]

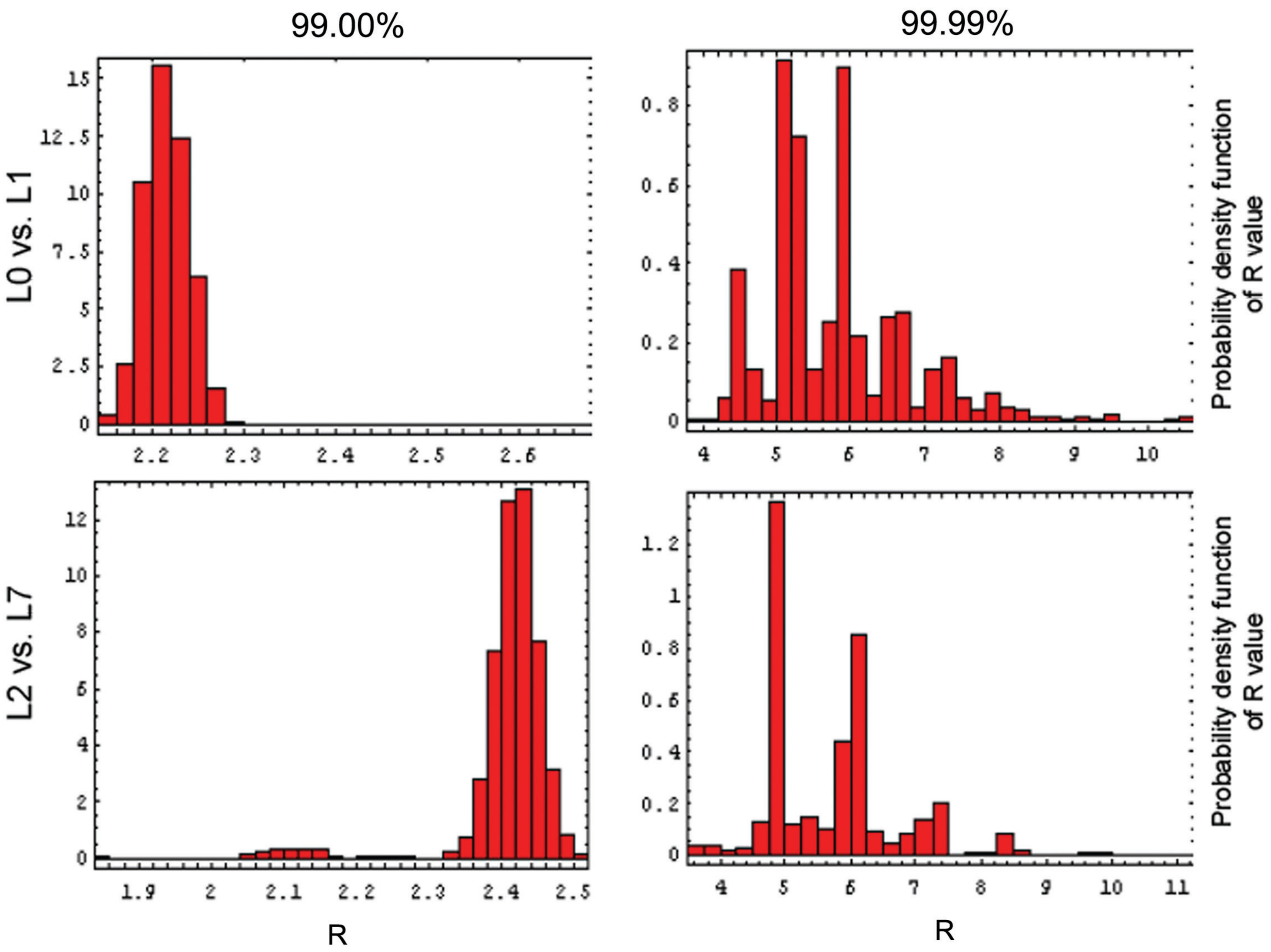

a

Comparison	Specificity	R-value Cut offs
L0 vs. L1	99,00%	2.21715
L0 vs. L1	99.99%	5.78486
L2 vs. L7	99,00%	2.40962
L2 vs. L7	99.99%	5.70563

b

Figure S2.
Calculation of the Statistical Significant Thresholds of the R-statistic at Different Specificities
(A) Distribution of the R-statistic for the indicated specificities and pairwise library comparisons.
(B) Tabulated R-value cutoffs for the indicated specificities and pairwise library comparisons.
