# Supplementary figures and images for: Cytoskeletal Rearrangements in Synovial Fibroblasts as a Novel Pathophysiological Determinant of Modeled Rheumatoid Arthritis
Source: PLoS Genet. 2005 Oct 28;1(4):e48. doi: 10.1371/journal.pgen.0010048 (PMC1270006; doi:10.1371/journal.pgen.0010048)

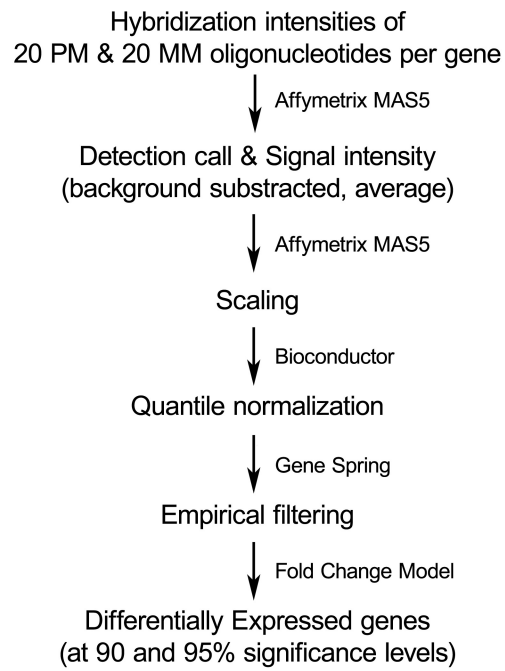

**Figure S3.**  
Microarray Data Normalization and Analysis Outline

Supplement: Figure S3 — (775 KB PDF) [file pgen.0010048.sg003.pdf]
