# Supplementary material for: Cytoskeletal Rearrangements in Synovial Fibroblasts as a Novel Pathophysiological Determinant of Modeled Rheumatoid Arthritis
Source: PLoS Genet. 2005 Oct 28;1(4):e48. doi: 10.1371/journal.pgen.0010048 (PMC1270006; doi:10.1371/journal.pgen.0010048)
Supplement: Figure S4 — (A) Quantile normalization on separated oligonucleotide chip subunits. (B) Reproducibility of technical duplicate samples in Affymetrix hybridizations. (C) “Sample type”-specific FCMs derived from replicated chips. (2.2 MB PDF) [file pgen.0010048.sg004.pdf]

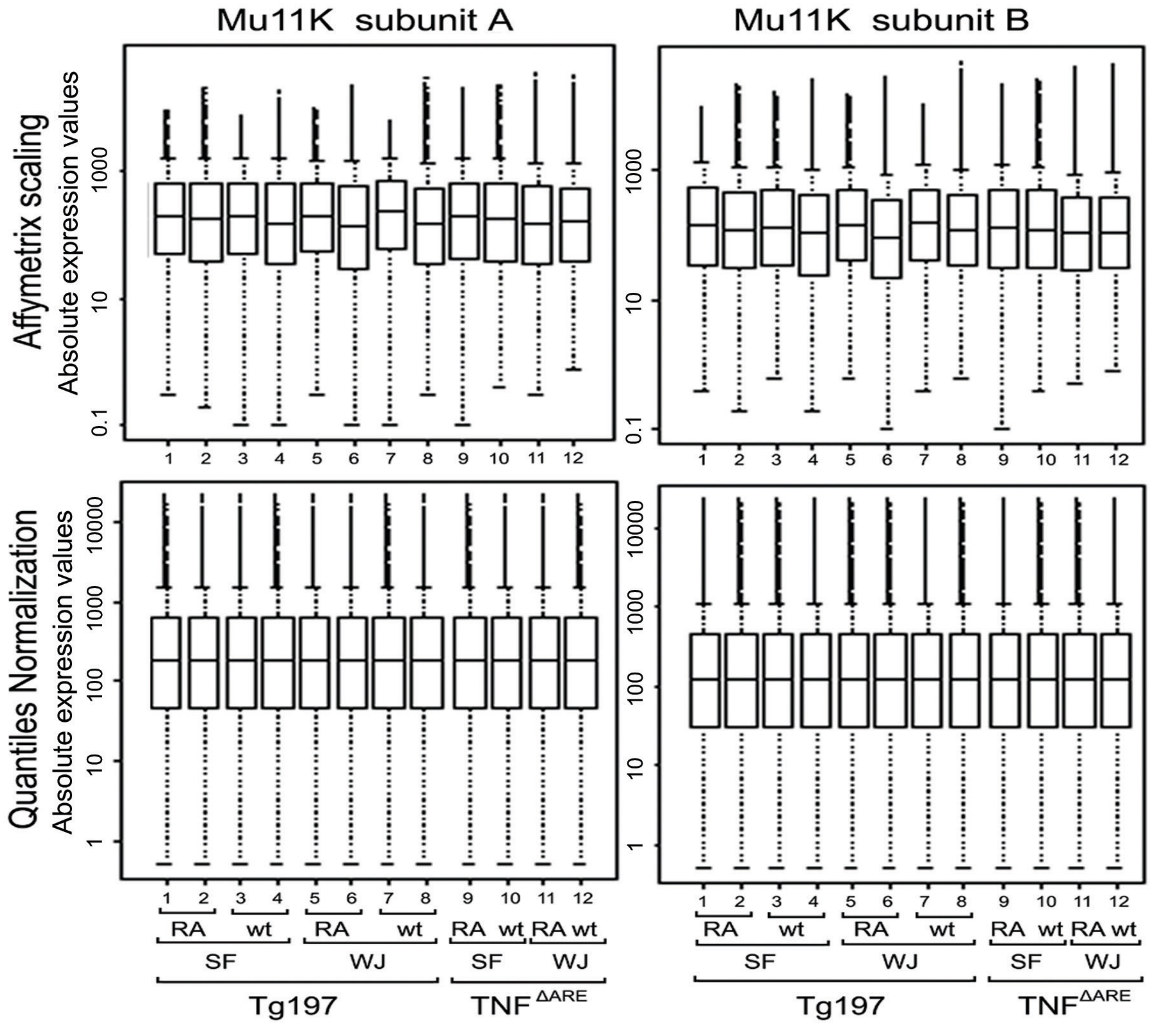

a

	Pearson	Detection
**Tg197 pSFs**	0,863	89,6
**wt pSFs**	0,887	90,6
**Tg197 WJs**	0,910	86,9
**wt WJs**	0,841	87,3

b

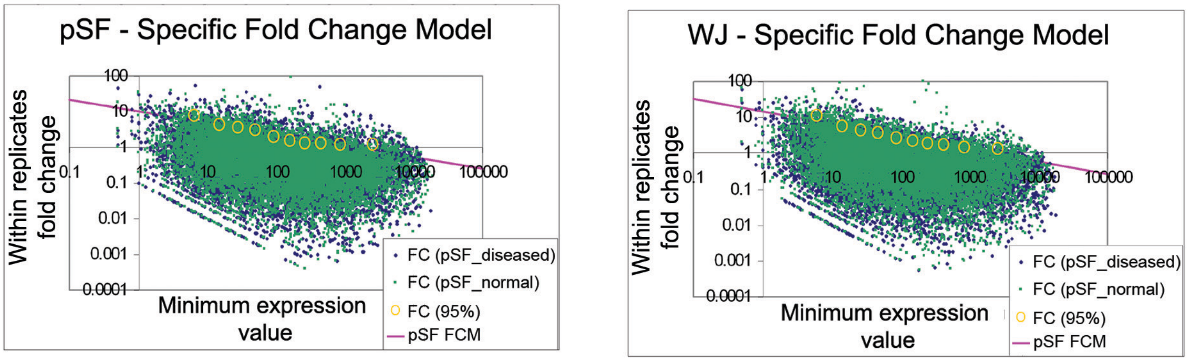

c

Figure S4.
Microarray Data Normalization, Evaluation, and Statistical Selection
(A) Quantile normalization on separated oligonucleotide chip subunits.
(B) Reproducibility of technical duplicate samples in Affymetrix hybridizations.
(C) “Sample type”-specific FCMs derived from replicated chips.
