# Supplementary material for: Cytoskeletal Rearrangements in Synovial Fibroblasts as a Novel Pathophysiological Determinant of Modeled Rheumatoid Arthritis
Source: PLoS Genet. 2005 Oct 28;1(4):e48. doi: 10.1371/journal.pgen.0010048 (PMC1270006; doi:10.1371/journal.pgen.0010048)
Supplement: Figure S5 — The associations were identified through the Biolab Experiment Assistant text-mining software. (A) PubMed identification numbers of corresponding publications. (B) Schematic representation of text mining results. Red and green indicate up-regulated and down-regulated genes, respectively; black and blue indicate WJ and SF, respectively; numerical values indicate number of PubMed references. (1.5 MB PDF) [file pgen.0010048.sg005.pdf]

	GENES	PUBMED ID
WJ	*Mb*	1293587, 8125137, 392694, 3467425
	Bsg	1634773
	Pstpip1	11971877
	Ctss	11856830, 11733367, 11920402
	Cd14	1930331, 11801661, 2504878, 3260544, 1713188, 1571092, 11549372, 10686511, 1378495, 7679058, 8162637, 8012331, 12571842, 1691032, 12746896, 11812022, 10685794, 11958435, 12384915, 1309559, 7526870, 12417058, 10562301, 1616358, 8546720, 8546719, 8849348, 11014344, 9811059, 1380987, 8568271, 8912515, 7686370, 9851271, 8976638, 9849315, 7561064, 8316769, 7562767, 9281388, 10614777, 15020327, 12672180, 10219258, 8639181, 12070677, 9592864, 14749527, 15034063, 14730603, 12949960, 15142274, 14607961, 9048866, 15146415, 12115225, 12375337, 15158620, 15140775, 15196219, 2575080, 9058650, 14632917, 10901283
	Ncf1	15936744, 15081107, 12935777, 12869136, 12546136, 12461526
	Ccl9	15905581
	Cstb	10025098
	Grn	10860865
	Timp1	10765925, 11097207, 8015285, 11062605, 11035124, 10990217, 11248661, 10914841, 12005365, 11040455, 11561108, 8080738, 8086445, 8129774, 12237470, 12296867, 12296865, 10943872, 11824946, 12051403, 11665967, 12571631, 11147173, 11762950, 11371663, 7808967, 8137899, 12379519, 10693865, 12508384, 12447632, 12447637, 12879774, 12509619, 12011369, 11508425, 12139676, 12682616, 8546529, 8849537, 8651985, 11710706, 12586482, 7880117, 7612046, 12939814, 10724258, 10752493, 8921246, 10888709, 12009332, 11792884, 9297577, 9049977, 8877931, 8919199, 9396371, 10415727, 9177919, 7490368, 12810425, 10365570, 9704635, 9704636, 7639798, 9645358, 12616343, 9972954, 9236673, 15040007, 9918237, 10025098, 12945803, 14611107, 15320915, 14760791, 15194590, 14740449, 9232430, 12913922, 8814070, 14523226, 10807502, 14705229, 10513800, 9513609, 14696674, 10880256, 15130753, 11399100, 15229943, 10397973, 10388526, 14714889, 9344881, 15144129, 10834863, 10464548, 12858344
SF	*Hp*	3462903, 7534965, 109055, 7688480, 749768, 9881748, 15146432
	Saa3	11160347, 2928311
	C3	6719058, 6723125, 3904645, 3904889, 3484437, 3484727, 2935508, 3954461, 3954803, 2420896, 3698338, 3084278, 3518030, 3486637, 2423091, 3521439, 3487899, 3743149, 3638367, 3462903, 3530991, 3532285, 3766605, 3778542, 2946496, 2946590, 3782971, 3538345, 3788578, 3789816, 3791699, 3823794, 3552978, 3579385, 3495479, 2820320, 2443529, 3668974, 2823368, 3499707, 3499907, 3689000, 3500676, 3423618, 3501355, 2963127, 3257873, 2830890, 3442962, 2833185, 3259803, 2837251, 3133151, 3389208, 3261029, 3058055, 3264781, 3065568, 2650989, 2785142, 2785367, 2469949, 2500276, 2741472, 2472921, 2474900, 2764813, 2790893, 2477604, 2797372, 2805423, 2810276, 2582690, 2591122, 2613159, 2624928, 2319523, 2158476, 2339901, 2111124, 2346005, 2349438, 2191409, 2141558, 1974926, 1697427, 2401118, 2170287, 2241286, 2146995, 2147105, 1991211, 1996481, 2077471, 1706901, 2024711, 2068540, 2070569, 1872046, 1680256, 1657009, 1657271, 1747138, 1768157, 1732316, 2980129, 1839528, 1316744, 1593589, 1595002, 1632661, 1395130, 1464075, 1484414, 1295608, 1307351, 1285067, 8230010, 8252159, 8267029, 8269781, 1285262, 8310205, 8111198, 8208996, 8055195, 8085060, 7939135, 7955597, 7860508, 7870343, 7534965, 1366144, 7548882, 10725071, 10759759, 10911799, 10937386, 11052177, 11125324, 11149553, 11174137, 11244314, 11315937, 11352263, 11354304, 11354563, 11550967, 11857338, 11953970, 12063122, 12077276, 12139377, 12142347, 12212115, 12220103, 12453313, 12475006, 12514522, 12682268, 12695150, 12723199, 12871189, 7702398, 7747149, 7774064, 7621584, 8705012, 8553595, 8572737, 8740816, 8742620, 8850166, 8902492, 9038384, 9093774, 9134821, 9150088, 9256031, 9402863, 9471394, 9472668, 9548303, 9550483, 9558172, 9676177, 9706420, 9782535, 9794428, 9805851, 9841702, 10423787, 10487137, 10510406, 5059642, 4114708, 4796805, 1078825, 1092716, 806270, 806271, 50336, 808229, 169295, 1099964, 1185743, 1081730, 1202608, 1215899, 1082751, 1277573, 819459, 950631, 961078, 786171, 61163, 970989, 980923, 825053, 830776, 1022870, 849359, 853126, 857743, 140691, 301046, 327037, 881695, 302538, 578869, 919557, 921822, 922113, 597373, 412488, 338220, 591304, 339851, 145832, 629093, 415670, 659731, 307274, 675210, 278177, 99092, 82340, 718271, 364409, 364609, 364636, 105740, 374144, 155958 443249, 109097, 312646, 377474, 114636, 315208, 40532, 315842, 517464, 93146, 392746, 7352929, 526442, 394545, 119858, 7360040, 7362682, 7389202, 7415585, 6774672, 6158571, 6903558, 6159620, 7441656, 6160859, 7459014, 7221787, 6908792, 6939879, 6788011, 6788211, 6166689, 6789016, 7258747, 7024339, 6456623, 7027433, 6794352, 7306228, 6914866, 7035034, 7337970, 6978407, 7073345, 7340719, 6210468, 6978853, 7085116, 7092168, 6212656, 7099337, 7100806, 7051254, 6981226, 6981473, 6981475, 6288942, 7125712, 6982590, 6983116, 7174786, 6184771, 7178855, 7160109, 6339939, 6339966, 6220848, 6221484, 6601932, 6342551, 6847725, 6857174, 6602812, 6603297, 6409476, 6879093, 6879095, 6411401, 6883809, 6684168, 6616816, 6623009, 6628574, 6316601, 6606402, 6418086, 6652392, 6608359, 6608422, 6201023, 6324537, 6712297, 12761187, 12902784, 14748705, 14768941, 14987271, 12811508, 15067519, 15054157, 15241561, 15320901, 15310311, 6375688, 6376381, 6611139, 6380835, 6381253, 6384505, 6384506, 6333061, 6333715, 6392549, 6514386, 6520829, 6395323, 3918543, 3158395, 3892637, 4017290, 3849467, 3875589, 4045842, 4048760, 4050141, 3876836, 2932466
	Mmp3	9892501, 15142265, 14872511
	Mmp13	10753945, 9892501
	Timp3	10765925, 12571631, 14532148, 9794437, 12296867, 11207308, 12237470, 12005365
	Aqp1	15024704, 12746451
	Eef1a1	10922075
	Tuba1	11048656, 9562601, 9022114, 1776991

a

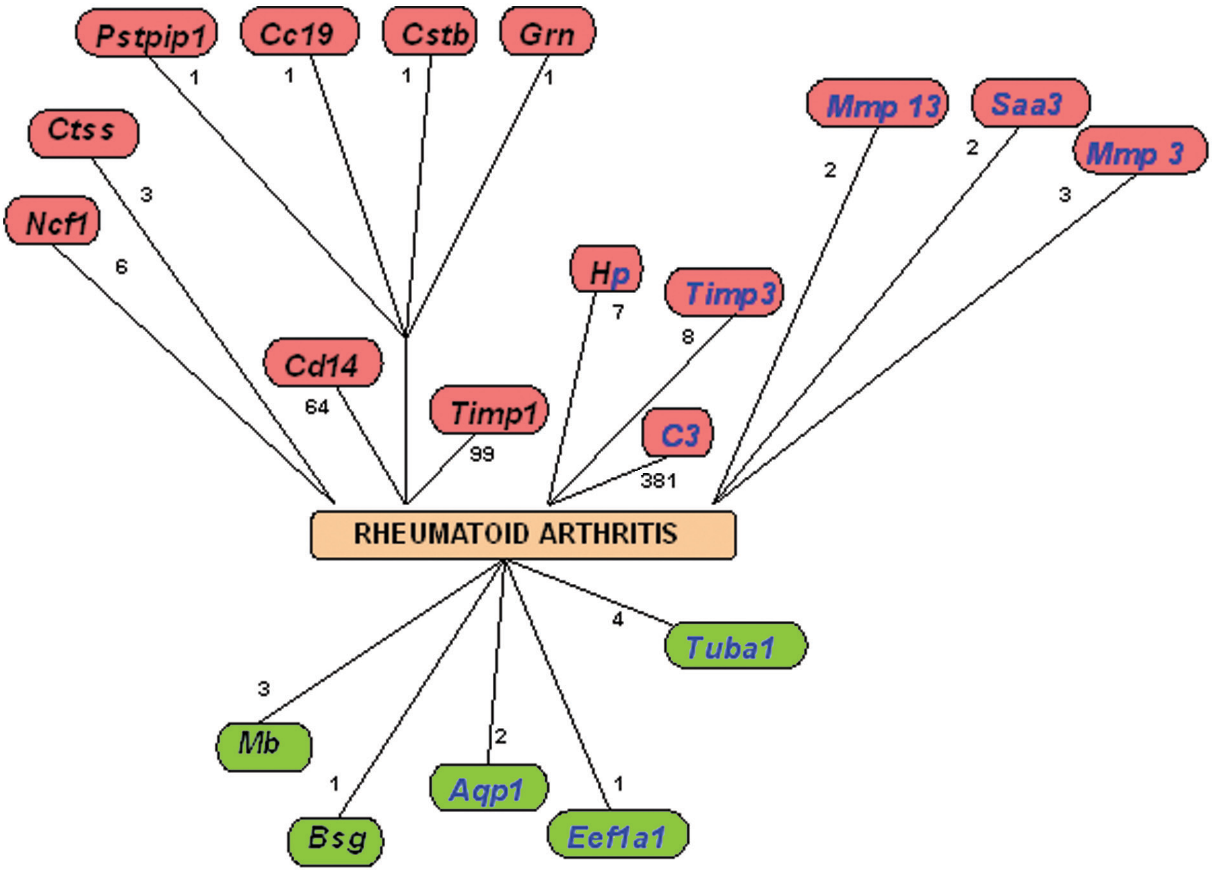

b

Figure S5.
Deregulated Genes Previously Reported to Be Associated with RA
The associations were identified through the Biolab Experiment Assistant text-mining software.
(A) PubMed identification numbers of corresponding publications.
(B) Schematic representation of text mining results. Red and green indicate up-regulated and down-regulated genes, respectively; black and blue indicate WJ and SF, respectively; numerical values indicate number of PubMed references.
