# Supplementary material for: Cytoskeletal Rearrangements in Synovial Fibroblasts as a Novel Pathophysiological Determinant of Modeled Rheumatoid Arthritis
Source: PLoS Genet. 2005 Oct 28;1(4):e48. doi: 10.1371/journal.pgen.0010048 (PMC1270006; doi:10.1371/journal.pgen.0010048)
Supplement: Figure S6 — (A–C) SQ RT-PCR of the indicated genes from different cDNA amounts of arthritic or WT WJs, primary SFs (pSF) and immortalized SFs (iSF). Ethidium bromide-stained PCR products were quantified with GelWorks 1D Advanced (v. 4.01) software and were normalized against the expression of B2M. (D) Real-time RT-PCR of the indicated genes from the indicated samples. (E) Western blot of whole cell extracts (two independent preparations) from arthritic and WT immortalized SFs probed with antibodies to gelsolin and actin. Gelsolin immunostaining was quantified with GelWorks 1D Advanced (v. 4.01) software and normalized against the corresponding actin intensities. (2.0 MB PDF) [file pgen.0010048.sg006.pdf]

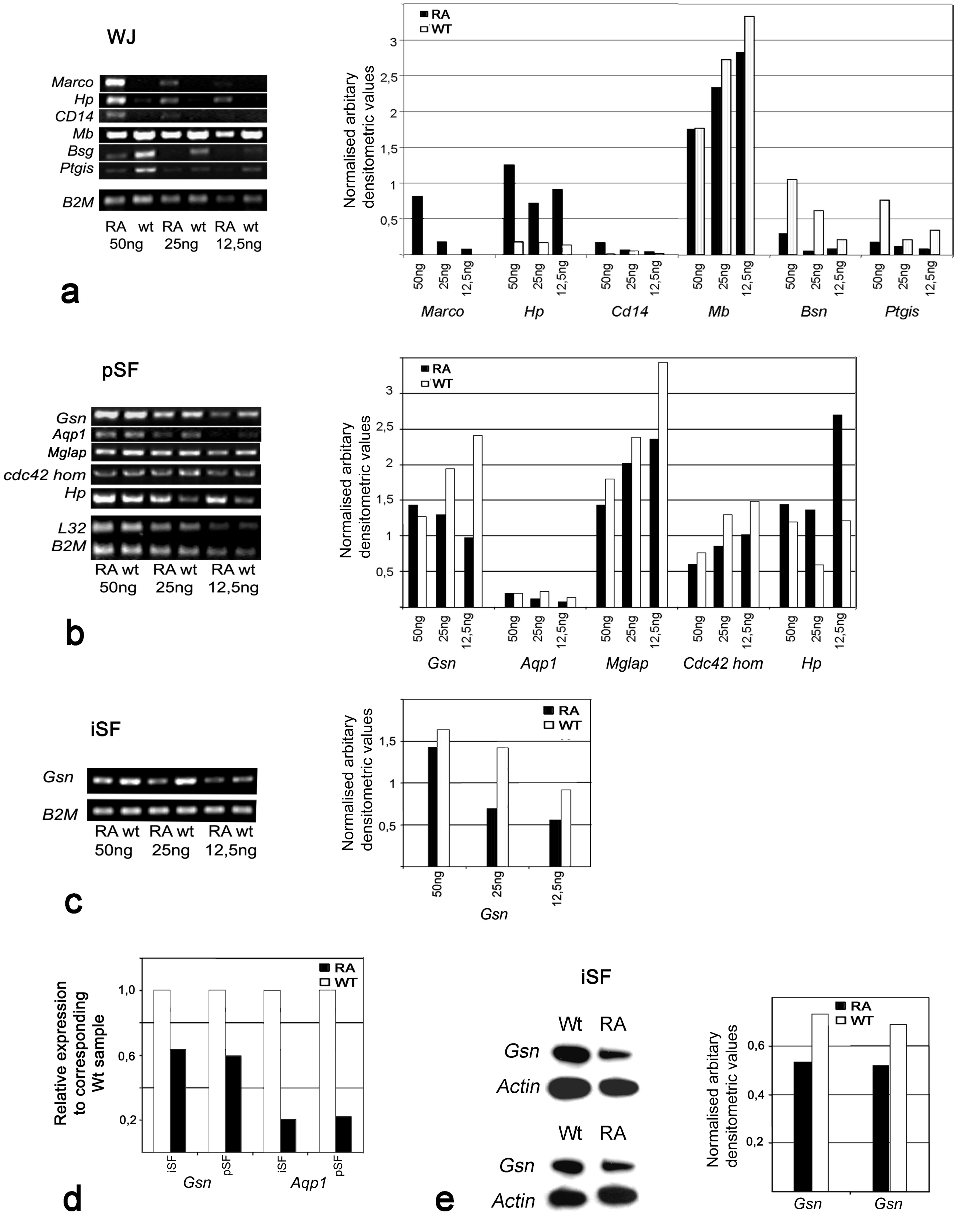

Figure S6.
Validation of Expression Profiling Results
(A–C) SQ RT-PCR of the indicated genes from different cDNA amounts of arthritic or WT WJs, primary SFs (pSF) and immortalized SFs (iSF). Ethidium bromide-stained PCR products were quantified with GelWorks 1D Advanced (v. 4.01) software and were normalized against the expression of B2M.
(D) Real-time RT-PCR of the indicated genes from the indicated samples.
(E) Western blot of whole cell extracts (two independent preparations) from arthritic and WT immortalized SFs probed with antibodies to gelsolin and actin. Gelsolin immunostaining was quantified with GelWorks 1D Advanced (v. 4.01) software and normalized against the corresponding actin intensities.
